# How neuroticism affects prejudical attitudes and social distance

**DOI:** 10.1192/j.eurpsy.2021.1178

**Published:** 2021-08-13

**Authors:** J. Jonáš, R. Heissler, N. Doubková, M. Preiss

**Affiliations:** 1 Clinical Research Of Mental Disorders, National Institute of Mental Health, Klecany, Czech Republic; 2 Faculty Of Arts, Charles University, Prague, Czech Republic; 3 Faculty Of Education, Charles University, Prague, Czech Republic; 4 Psychology, University of New York in Prague, Praha, Czech Republic

**Keywords:** racism, Neuroticism, prejudice, social distance

## Abstract

**Introduction:**

Previous studies didn’t find any connection between Neuroticism and authoritarian personality or social dominance orientation, but xenophobic attitudes might be hold even apart from these constructs.

**Objectives:**

In our study we compared subjects with high Neurtoticism score with controls with a focus on racism and social distance.

**Methods:**

The Bogardus Social Distance Scale (BSDS) is a measure of perceived social distance of a subject towards concrete outgroups. Modern Racism Scale (MRS) and The Blatant and Subtle Prejudice Scales (BSRS) are scales measuring racism. For our study we used the Neuroticism scale of the Eysenck Personality Questionnaire (EPQ). Also, we asked about personal conflicts with outgroup members and how subjects perceived their unpleasantness and importance. We measured the attitudes towards Romani, Vietnamese, foreigners, homeless people, migrants, people with mental disease and people with a physical disability.

**Results:**

People with the high neuroticism score (SD≥1; N=48) showed significantly higher scores in racism. In comparison to control group (CG; N=96), their social distance differed significantly towards Romani, Vietnamese and migrants. Effect sizes were however on the threshold between weak and moderate. After Bonferroni correction, only the social distance towards migrants remained significant. People with the high neuroticism score didn’t report higher rate in conflict with outgroup members than HC.

**Conclusions:**

People with the high neuroticism score showed different pattern in attitudes towards outgroup members, but not in conflict with them, which might point at higher need of internalization of negative attitudes.

